# Fibroblast Growth Factor 23 and Osteoporosis: Evidence from Bench to Bedside

**DOI:** 10.3390/ijms23052500

**Published:** 2022-02-24

**Authors:** Wachiranun Sirikul, Natthaphat Siri-Angkul, Nipon Chattipakorn, Siriporn C. Chattipakorn

**Affiliations:** 1 Department of Community Medicine, Faculty of Medicine, Chiang Mai University, Chiang Mai 50200, Thailand; wachiranun.sir@cmu.ac.th; 2 Neurophysiology Unit, Cardiac Electrophysiology Research and Training Center, Faculty of Medicine, Chiang Mai University, Chiang Mai 50200, Thailand; natthaphat.s@cmu.ac.th (N.S.-A.); nipon.chat@cmu.ac.th (N.C.); 3 Cardiac Electrophysiology Unit, Department of Physiology, Faculty of Medicine, Chiang Mai University, Chiang Mai 50200, Thailand; 4 Center of Excellence in Cardiac Electrophysiology Research, Chiang Mai University, Chiang Mai 50200, Thailand; 5 Department of Oral Biology and Diagnostic Sciences, Faculty of Dentistry, Chiang Mai University, Chiang Mai 50200, Thailand

**Keywords:** FGF23, osteoblast, osteoclast, biochemical markers, bone remodeling, osteoporosis

## Abstract

Osteoporosis is a chronic debilitating disease caused by imbalanced bone remodeling processes that impair the structural integrity of bone. Over the last ten years, the association between fibroblast growth factor 23 (FGF23) and osteoporosis has been studied in both pre-clinical and clinical investigations. FGF23 is a bone-derived endocrine factor that regulates mineral homeostasis via the fibroblast growth factor receptors (FGFRs)/αKlotho complex. These receptors are expressed in kidney and the parathyroid gland. Preclinical studies have supported the link between the local actions of FGF23 on the bone remodeling processes. In addition, clinical evidence regarding the effects of FGF23 on bone mass and fragility fractures suggest potential diagnostic and prognostic applications of FGF23 in clinical contexts, particularly in elderly and patients with chronic kidney disease. However, inconsistent findings exist and there are areas of uncertainty requiring exploration. This review comprehensively summarizes and discusses preclinical and clinical reports on the roles of FGF23 on osteoporosis, with an emphasis on the local action, as opposed to the systemic action, of FGF23 on the bone. Current gaps in knowledge and future research directions are also suggested to encourage further rigorous research in this important field.

## 1. Introduction

Osteoporosis is a chronic debilitating disease caused by an imbalance in bone remodeling processes that favor bone resorption over bone formation. Structural integrity of the bone is maintained through intricate and interrelated activities of bone-forming osteoblasts, bone-resorbing osteoclasts, physicochemical conditions that modulate local matrix mineralization, and systemic mineral homeostasis. Over the last ten years, the association between fibroblast growth factor 23 (FGF23) and osteoporosis has been studied [1,2,3,4]. FGF23 is a bone-derived endocrine factor that regulates phosphate and vitamin D homeostasis via the fibroblast growth factor receptors (FGFRs)/αKlotho complex. These receptors are expressed in kidney and the parathyroid gland [5,6,7]. The FGF23-mediated mechanism interacts with the classical calcium/phosphate regulating processes driven by parathyroid hormone (PTH) and calcitriol (active vitamin D). In addition to the systemic effects of FGF23, preclinical studies have revealed mechanistic insights in the local actions of FGF23 on bone remodeling processes [8,9]. Moreover, accumulating evidence from clinical studies reported the association between FGF23, bone remodeling and fragility fracture in the elderly, either with or without a decline in renal function. These recent advances in the insights regarding FGF23 effects in mineral homeostasis and bone remodeling suggest potential clinical applications of FGF23 in the clinical context. However, inconsistent findings exist and there are areas of uncertainty requiring an exploration. Thus, this review comprehensively summarizes preclinical and clinical reports of the roles of FGF23 on osteoporosis and chronic kidney disease–mineral and bone disease (CKD-MBD), with an emphasis on the local actions, as opposed to systemic actions, of FGF23 on the bone. The potential use of FGF23 as a biomarker for osteoporosis, CKD-MBD and fragility fracture prediction is also discussed. Finally, current gaps in knowledge and future research directions are also suggested to encourage further rigorous research in this important field.

## 2. Regulation of FGF23 Expression

FGF23 is mainly produced by osteoblasts and osteocytes [10,11,12]. In those cell types, FGF23 expression is stimulated by the calcitriol (via the vitamin D receptor [VDR] signaling pathway) and hyperphosphatemia (via the sensing of extracellular phosphate levels mediated by type III sodium-dependent phosphate transporter [PiT2]) [9,13] and FGFR1c [14]. There are two possible mechanistic links of high extracellular phosphate (Pi) regulating FGF23 secretion via FGFR1c and PiT2 actions. For the first hypothesized mechanism, Pi-dependent stimulation of the ERK/MAPK pathway via FGFR1c can promote GALANT3 expression, preventing intact FGF23 (iFGF23) cleavage by O-glycosylation [14]. However, the mechanisms of FGFR1c activation by extracellular phosphate remain unclear. Another study proposed the mechanism in which high extracellular phosphate acts on osteocytes/osteoblasts via the PiT2 sensors, increasing iFGF23 levels in FAM20C (promote FGF23 cleavage by phosphorylation at cleavage site) and GALANT3 independent mechanisms [13]. Furthermore, there was no significant change in Pi-dependent FGF23 secretion when the ERK/MAPK pathway was inhibited. It is possible to conclude that the physiological link between Pi-dependent activation and FGF23 secretion is mostly dependent on PiT2 action through other downstream signaling pathways other than ERK/MAPK. As discussed, the mechanistic understanding of Pi-dependent FGF23 regulation via PiT2 sensing remains to be explored.

## 3. FGF23 Effects on Mineral Homeostasis

**Figure 1:**
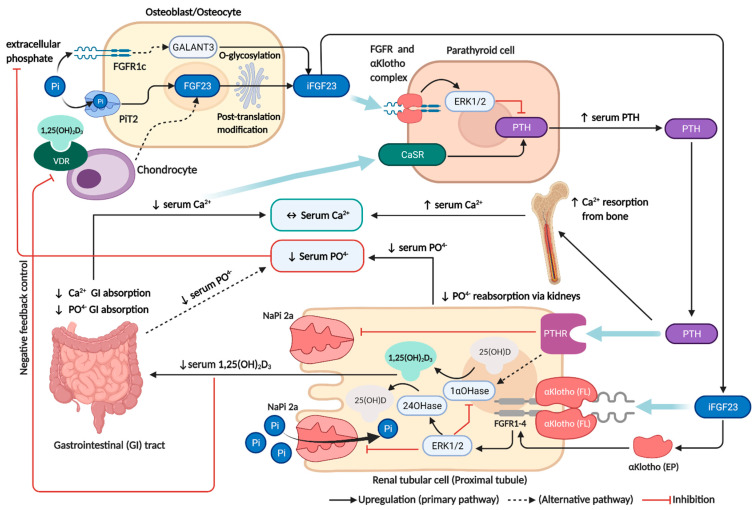
FGF23 regulation and its interaction with the traditional paradigm on mineral homeostasis. FGF23 is mainly produced by osteoblasts and osteocytes, which is stimulated by calcitriol via the VDR signaling pathway and hyperphosphatemia via sensing of extracellular phosphate levels mediated by PiT2 (dominant pathway) and FGFR1c. In proximal tubular cells, FGF23 binding with the αKlotho/FGFR complex causes hypophosphatemia by inhibiting NaPi-2a and decreased calcitriol levels by suppressing the vitamin D activation process. Decreased calcitriol also reduces calcium and phosphate absorption from the GI tract. In parathyroid cells, PTH secretion is dominantly controlled by circulating calcium via CaSR, which overrides the inhibitory effect of FGF23. Increased PTH induces calcium resorption from bone and also causes hypophosphatemia by inhibiting NaPi-2a activity. Consequently, hypophosphatemia and decreased calcitriol levels will act as the negative feedback control of FGF23 production by diminishing VDR, PiT2 and FGFR1c signaling. This figure was generated with publication licensed by BioRender, Toronto, ON, Canada (Agreement number: VZ237SOI81, 19 November 2021). Abbreviations: CaSR, Calcium-sensing receptor; EP, Extracellular αKlotho; ERK1/2, Extracellular signal-regulated kinases; FL, Full-length αKlotho; FGF23, Fibroblast growth factor 23; FGFR, Fibroblast growth factor receptor; GALNT3, polypeptide N-acetyl.

Circulating FGF23 acts on the FGFRs/αKlotho complex (particularly those containing the FGFR subtypes 1, 3, or 4) on renal proximal tubular cells [6], and parathyroid cells [7,15]. In the kidney, increasing FGF23 levels causes hypophosphatemia and decreased calcitriol levels. In the proximal tubules, FGF23 reduces phosphate reabsorption by suppressing the expression of sodium-dependent phosphate co-transporter type II a (NaPi-2a) [5,6,9]. Vitamin D metabolism is also under tight regulation by FGF23, which downregulates 1α-hydroxylase and upregulates 24-hydroxylase in the proximal tubular cells [5,6,9]. Consequently, hypophosphatemia and decreased calcitriol levels inhibit bone derived FGF23 secretion as a negative feedback control by diminishing VDR and extracellular phosphate signalling in the bone. In the parathyroid gland, PTH regulation is another important pathway for bone-mineral homeostasis. The suppression of PTH by FGF23 is primarily mediated by a Klotho-dependent mechanism through the MAPK/ERK signalling pathway [7,15] and Klotho-independent mechanism via the calcineurin signalling pathway [16]. Despite the fact that FGF23 significantly inhibits PTH production and secretion, the dominant regulators of PTH levels remain at circulating free (ionized) calcium and calcitriol levels, which are monitored by the calcium-sensing receptor (CaSR) and the VDR in the parathyroid gland [17,18]. Subsequently, low calcitriol levels and hypocalcemia induced by increasing FGF23 levels indirectly stimulate PTH synthesis and release in the parathyroid via VDR and CaSR, overriding the inhibitory action of FGF23. Thus, elevated PTH can counterbalance the calcium-lowering effect of FGF23 by increasing calcium resorption from bone [5]. The physiological actions of FGF23 on mineral homeostasis are summarized in Figure 1 and Supplementary Materials Table S1.

Given the fact that human mineral homeostasis is mostly dependent on the kidney, loss of renal function in CKD and ESRD has a significant impact on mineral homeostasis, including hyperphosphatemia, hypocalcemia, and lowered calcitriol. Subsequently, FGF23 levels rise early and steadily with the progression of kidney function in the early stages of CKD as a physiologic compensation to maintain normal phosphorus balance by enhancing urinary phosphate excretion in conjunction with indirectly increased parathyroid hormone levels and decreasing gut phosphorus and calcium absorption through decreased calcitriol synthesis. In the late stages, this compensatory mechanism may become maladaptive from the loss of renal functions, resulting in a progressive increased FGF23 level, decreased calcitriol levels, hypocalcemia, and associated consequences such as CKD-MBD from a secondary hyperparathyroidism. Excess FGF23 levels in CKD/ESRD also contribute directly to bone remodelling and mineralization. The details of FGF23 effects on the pathogenesis of CKD-MBD will be discussed later.

## 4. Local Effects of FGF23 on Osteoblast and Bone Formation

Previous experimental studies have demonstrated that bone-derived FGF23 affects bone remodeling. Under physiologic conditions, an in vitro study of mouse osteoblasts showed that increased FGF23 protein expression was associated with peaked osteoblastic activity and increased ALP bone nodules. During the same period, increased OPN expression was also observed that might relate to increased FGF23 [10]. This study also showed that increasing calcitriol caused FGF23 upregulation and matrix mineralization inhibition in a dose-dependent fashion. This result raises the possibility that FGF23 regulates bone mineralization by controlling OPN expression. These findings were consistent with results from another in vitro study which showed that treating mouse MSCs with a physiologic concentration of FGF23 promoted osteoblast differentiation and activity by up-regulating OC and ALP, and OPN in a dose-dependent manner [12]. Inhibition of the FGF23-FGFRs-αKlotho pathway caused diminished osteoid nodule formation, reduced expression of osteoblast markers, and OPN. This finding supports the existence of an auto-/paracrine effect of FGF23 via the FGFRs-Klotho complex.

**Figure 2:**
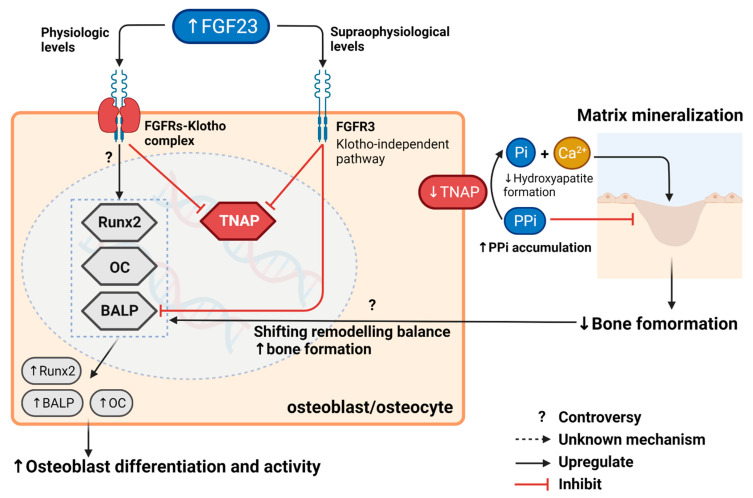
Regulation of FGF23 and its autocrine/paracrine effects on bone formation. In supra physiologic conditions, FGF23 acts directly on FGFR3 in a Klotho-independent manner, thereby inhibiting bone formation. Increased FGF23 suppresses differentiated osteoblast activity and TNAP transcription, which subsequently causes PPi accumulation in the ECM and inhibits matrix mineralization. In physiological conditions, the actions of FGF23 on canonical receptors (FGFRs-Klotho complex) also downregulate TNAP, decreasing matrix mineralization. However, the upregulation of osteoblastic markers in these conditions may be caused by the shifting of remodelling balance toward bone formation or direct action of FGF23 via canonical receptors. The symbol “?” and dash lines denote issues of controversy and unknown mechanisms, respectively. This figure was generated with publication licensed by BioRender, Toronto, ON, Canada (Agreement number: VC237SOKSX, 19 November 2021). Abbreviations: BALP, Specific bone Alkaline phosphatase; FGF23, Fibroblast growth factor 23; Pi, Inorganic phosphate; PPi, Pyrophosphate; Runx2, Runt-related transcription factor 2; TNAP, Tissue nonspecific alkaline phosphatase; OC, Osteocalcin.

According to recent preclinical evidence, auto-/paracrine effects of FGF23 on bone mineralization have been uncovered in Klotho and in a calcitriol independent manner [19]. FGF23 action on FGFR3 indirectly regulated bone mineralization by suppressing tissue nonspecific alkaline phosphatase (TNAP), whereas calcitriol-VDR action directly increased OPN transcription in osteoblasts. This study revealed the mechanistic insight of FGF23 impacts on bone mineralization through two key players: pyrophosphate (PPi) and inorganic phosphate, but not directly via OPN transcription. According to well-established evidence, PPi is synthesized intracellularly by ectonucleotide pyrophosphatase/phosphodiesterase 1 and 3 (ENPP1 and ENPP3) [20], then transported to the extracellular matrix (ECM) via the transmembrane protein ANK [21]. PPi can inhibit hydroxyapatite crystal formation and deposit on type I collagen [22,23]. Increased levels of PPi in the ECM therefore inhibit, bone mineralization through the same mechanism as OPN activity [24]. Consequently, PPi in the ECM is hydrolyzed into inorganic phosphate by tissue nonspecific alkaline phosphatase (TNAP) [25]. Inorganic phosphate is a major component of hydroxyapatite crystals and an established stimulator of OPN secretion by an unknown mechanism [26]. In this study, the supraphysiological concentration of FGF23 showed an inhibitory effect on TNAP activity via the FGFR3-ERK pathway, thereby increasing extracellular PPi and decreasing extracellular inorganic phosphate concentration, consequently decreasing OPN expression regardless of Klotho status. Additionally, ALP activity of differentiated osteoblasts from both wild-type and Klotho knock-out mice was significantly suppressed by FGF23 in a dose-dependent manner. Nevertheless, the Klotho-independent FGF23 action was found only under the supra physiologic condition (20-times of physiological FGF23 level). The aforementioned study showed that treating mice MSCs with physiologic concentrations of FGF23 promoted osteoblast differentiation and activity by dose-dependently up-regulating OC and ALP, whereas the Klotho knock-out model did not alter the expression of osteoblastic biomarkers in the same experiment [12]. These contradictory results of FGF23 effects on osteoblast differentiation and activity could be explained by the following explanations. Increased osteoblast differentiation and activity at physiological FGF23 levels may be a response to the remodelling balance shifting toward bone formation as a result of decreased bone mineralization. Another possibility is that FGF23 has bimodal effects in physiological and supraphysiological conditions. In physiological circumstances, FGF23 may act via the FGFRs-Klotho complex to promote osteoblast differentiation and activity, whereas supraphysiological FGF23 levels decrease bone formation by inhibiting bone mineralization and osteoblast activities in a Klotho-independent pathway. These results suggest possible roles of FGF23 in bone formation, as illustrated in Figure 2 and summarized in Supplementary Materials Table S2.

## 5. Local Effects of FGF23 on Osteoclast and Bone Resorption

**Figure 3:**
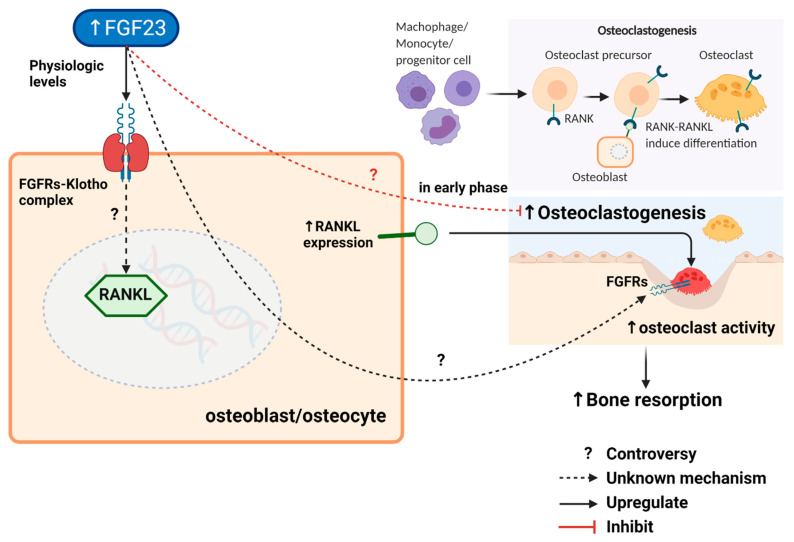
Regulation of FGF23 and its autocrine/paracrine effects on osteoclast and bone resorption. A decrease in osteoclastogenesis by downregulating RANKL expression may be mediated by FGF23 via an unknown mechanism. The biphasic physiological effects of FGF23 via FGFR on human monocyte-derived osteoclast cultures inhibit the early stages of osteoclastogenesis from osteoclast progenitors but substantially inhibited osteoclast-mediated bone resorption. However, the hypothesis that FGF23 may influence RANKL expression, osteoclastogenesis, and osteoclast-mediated bone resorption requires further confirmation. The symbol “?” and dash lines denote issues of controversy and unknown mechanism, respectively. This figure was generated with publication licensed by BioRender, Toronto, ON, Canada (Agreement number: NS237SOEQK, 19 November 2021). Abbreviations: FGF23, Fibroblast growth factor 23; RANK, Receptor activator of nuclear factor-κΒ; RANKL, Receptor activator of nuclear factor-κΒ ligand.

Two studies evaluated the association between FGF23 and osteoclasts [9,27]. From the first study [9], stimulating VDR signalling by adding calcitriol to the culture media in the VDR knock-out mouse chondrocyte/osteoblast co-culture model resulted in decreased FGF23 and an absence of RANKL expression without any alteration of other osteoblast markers (OC, Runx2, and OPG). In contrast, the wild-type model in the same experiment resulted in increased RANKL expression and non-significant increased FGF23. FGF23 expression was unchanged either in the wild-type or VDR knock-out osteoblast cultures. These findings suggest that chondrocyte VDR signalling regulates FGF23 expression, not osteoblast VDR. A decrease in osteoclastogenesis due to the lack of VDR signalling, thereby resulting in an increasing ratio between total bone volume and trabecular bone in this study, might be mediated by FGF23. However, the hypothesis that FGF23 may be a downstream effector of the chondrocyte VDR signalling pathway which influences RANKL expression requires further confirmation. The second in-vitro study directly investigated the association between FGF23 and osteoclasts in human monocyte-derived osteoclast cultures treated with FGF23 [27]. This study showed that biphasic physiological FGF23 effects via FGFR inhibited the early stages of osteoclastogenesis in human monocytes but marginally increased osteoclast-mediated bone resorption. However, the mechanistic link of local FGF23 actions on osteoclast biology remains unclear. These results suggest possible roles of FGF23 in bone remodelling by regulating osteoclastogenesis and osteoclast-mediated bone resorption, as illustrated in Figure 3 and summarized in Supplementary Materials Table S2. The mechanistic link between increased FGF23 and bone remodelling reported in these preclinical studies led to clinical investigations into the association between FGF23 and osteoporosis in the elderly, CKD or end-stage renal disease (ESRD) patients, who commonly have elevated levels of FGF23, as discussed later.

## 6. Role of FGF23 in Postmenopausal and Age-Related Osteoporosis Pathogenesis

Osteoporosis is primarily caused by an imbalance in the remodelling process. In the early stages of postmenopausal osteoporosis, a deficiency of estrogen induces an increase in RANKL expression, which results in increased osteoclast numbers and activity as well as concurrent suppression of osteoblast functions. Subsequently, increased net bone resorption outpacing bone formation caused rapid loss of mainly trabecular bone mass. As previously discussed, FGF23 inhibits bone mineralization by suppressing TNAP and resulting in PPi accumulation in both physiological and supraphysiological conditions. Thus, increased FGF23 could accentuate the net negative remodelling by inhibiting bone formation. Although some studies found that physiological concentration of increased FGF23 was associated with increased osteoblast differentiation and bone nodule formation, these might not be direct actions of FGF23 on osteoblasts but could be a compensatory response to the inhibition of bone formation. The auto-/paracrine inhibitory effect of FGF23 is also confirmed by the study that indicated that excessive levels of FGF23 decrease ALP activity in differentiated osteoblasts, whereas the absence of FGF23 signalling results in a Klotho-independent, cell-autonomous increase in ALP activity. Moreover, FGF23 may regulate the bone remodelling balance by inhibiting the early stages of osteoclastogenesis from osteoclast progenitors and promoting osteoclast-mediated bone resorption.

In the second longer phase (age-related osteoporosis), the impaired bone quality is the gradual loss of mineral from bones with aging, which are also influenced by other age-related conditions, such as deconditioning or frailty, vitamin D deficiency, and secondary hyperparathyroidism. An imbalance of systemic mineral homeostasis in the elderly particularly impaired renal function, hyperphosphatemia, and additionally led to an increase in FGF23 secretion, resulting in a progressive decrease in bone mineralization. Nevertheless, bone fragility and fragility fracture in osteoporosis are influenced by multiple risk factors including genetic features, the level of weight-bearing physical activity, nutrition, smoking, body mass index (BMI), concurrent diseases, and medications. The reported clinical evidence for an independent association between FGF23 and bone fragility in postmenopausal and age-related osteoporosis has been comprehensively summarized below.

## 7. The Association between FGF23 and Bone Fragility in the Elderly

As discussed previously, preclinical reports have demonstrated the possible effect of FGF23 on osteoporosis pathogenesis. Even though increased FGF23 showed the promising inhibitory effect on bone mineralization in preclinical studies, there was not enough supporting clinical evidence to establish a robust causal relationship between FGF23 and BMD, which is a major predictor of bone fragility. The large cohort of males with osteoporosis revealed only a weak association between FGF23 and bone mineral density (BMD) that diminished when adjusting for potential confounders, including, age, height, weight, and smoking [2]. Another study in men with osteoporosis also showed that femoral neck BMD was not significantly changed in different FGF23 quartiles [28]. In contrast, the studies in post-menopausal women found that FGF23 had a strong negative correlation with BMD [1] and a significant association with femoral BMD after adjusting for PTH, 25(OH)D, and leptin [4]. Furthermore, another cross-sectional study in osteoporosis patients, in which the majority are women, demonstrated a weak independent association between high FGF23 and deceased trabecular bone microarchitecture but not cortical bone [3]. However, this evidence was insufficient to prove that elevating FGF23 had a clinically significant effect on decreasing BMD in women. The paucity of the independent association between FGF23 level and age-related osteoporosis suggests that an autocrine/paracrine effect of FGF23 does not play a significant role in the pathogenesis of age-related osteoporosis. The clinical findings in the elderly are summarized in Table 1.

## 8. Role of FGF23 in CKD-MBD Pathogenesis

Human mineral homeostasis largely depends on the kidney. Hyperphosphatemia, hypocalcemia, and reduced calcitriol are caused by the loss of renal functions in CKD and ESRD, which leads to secondary hyperparathyroidism and CKD-MBD [32]. The emergence of FGF23 has reformed the understanding of the mechanisms underlying the development of secondary hyperparathyroidism [33]. The loss of FGF23 ability to regulate phosphate levels through its phosphaturic effect and inhibit PTH secretion was shown in ESRD patients, resulting in hyperphosphatemia and increasing FGF23 levels. Although FGF23 can directly block PTH synthesis and secretion from the parathyroid gland primary cells via both the Klotho/FGFRs pathway [7] and the Klotho-independent pathway [16], it can also indirectly induce hyperparathyroidism. In CKD and ESRD patients, increased FGF23 induced by hyperphosphatemia inhibits vitamin D activation and subsequently decreases calcium absorption in the intestine [34,35]. As a result, low calcitriol levels and hypocalcemia promote PTH synthesis and release via VDR and CaSR in parathyroid, overriding the inhibitory effect of FGF23 [18]. The progressive kidney dysfunction will subsequently cause hyporesponsiveness of VDR [36] on the parathyroid gland with more excessive PTH synthesis and reduced expression of CaSR [37] on the parathyroid gland leading to parathyroid gland hyperplasia and will become autonomous [38]. This excess PTH leads to further calcium resorption from the bone, resulting in abnormalities of bone architecture. Consequently, the auto-/paracrine effects of FGF23 on bone cells, mediated by high levels of circulating FGF23, is another possible pathogenesis of CKD-MBD, in addition to secondary hyperparathyroidism. The recent preclinical study in the CKD model indicates that excessive FGF23 secretion driven by renal failure significantly inhibits bone mineralization via TNAP suppression and PPi accumulation [39]. FGF23 neutralization’s effects on bone quality had been explored in vivo for its therapeutic potential in CKD-MBD [40,41]. Anti-FGF23 treatment was found to significantly improve the bone quality in CKD mice by correcting the secondary hyperparathyroidism, and increased calcitriol levels, indicating that FGF23 is a key factor of CKD related bone diseases. Nonetheless, other studies in CKD animal models indicated that FGF23 neutralization exacerbates hyperphosphatemia and elevated serum calcitriol, resulting in an increased arterial calcification. Thus, the systemic mineral disturbances caused by FGF23 neutralization limit its benefit on bone quality in CKD-MBD, which certainly contributes to the increased risk of cardiovascular events and death.

## 9. The Association of FGF23 and CKD-MBD

Prior studies in patients with moderate renal impairment and kidney transplant patients [28,34,35,42,43] found similar results to those seen in the elderly. The levels of FGF23 in these individuals were not different from those in elderly people without CKD/ESRD. Furthermore, FGF23 levels in patients with CKD-MBD, did not demonstrate a significant relationship with BMD. Only one study assessed the relationship between FGF23 and bone remodelling activities in postmenopausal women with moderate renal impairment, but the relationship was not statistically significant [34]. In ESRD patients with low bone density, excessive FGF23 and increased levels of a bone formation marker (BALP) were reported [44]. Despite these findings, an unadjusted association between FGF23 and either bone remodelling markers or BMD was neither statistically nor clinically significant [34,42,43,44,45]. As a result, the present evidence is not sufficient to support the hypothesis that increased FGF23 independently causes CKD-MBD. A future investigation is needed to clarify the relationship between FGF23, bone microarchitecture, BMD, and bone remodelling biomarkers, which are adjusted for potential confounders (e.g., gender, BMI, sex hormones, PTH, and serum mineral levels), to establish the clinical potential of FGF23 in CKD-MBD. Data from the relevant studies in CKD/ESRD are summarized in Table 2.

## 10. Potential Clinical Application of FGF23 in Osteoporosis and CKD-MBD

Although current clinical studies are insufficient to support those findings and the hypothesis that FGF23 is independently associated with bone mineralization decline in the elderly and CKD-MBD, several clinical studies show that FGF23 has been a potential predictor for fragility fractures. High circulating FGF23 levels were discovered to be an independent risk factor for overall fragility fracture in elderly men [29]. Furthermore, the three aforementioned studies discovered an independent association between elevated FGF23 and the incidence of fragility fractures in both moderate CKD and ESRD patients [28,34,35]. Even though FGF23 levels had an independent negative relationship with BMD in postmenopausal women [1,4], it was not an effective discriminator between osteopenia/osteoporosis and normal bone mass [4]. In addition, utilizing the FGF23 level for osteoporosis prediction in hemodialysis patients resulted in poor discrimination [44]. Considering that various fragility fracture prediction models (e.g., the FRAX score, Q Fracture) are based on well-established clinical predictors (e.g., gender, BMI, CKD, alcohol, smoking, and corticosteroid use), there are currently no models that incorporate clinical predictors describing both systemic and local bone mineral homeostasis. The growing evidence indicates that FGF23 can represent the status of systemic bone mineral balance, renal function, and probably bone remodelling, thus the value-added of FGF23 on fragility fracture prediction awaits upcoming studies to explore its clinical potential to improve the prognostication of osteoporosis and CKD-MBD patients.

## 11. FGF23 Measurement in Routine Clinical Practices

**Figure 4:**
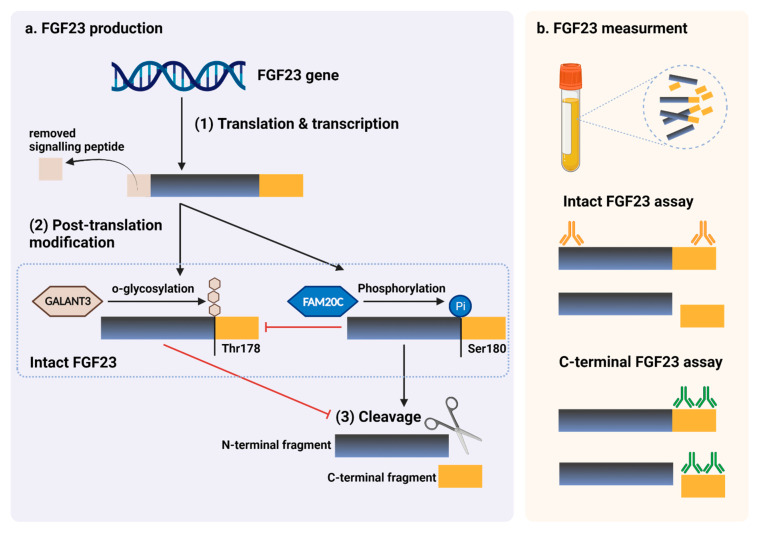
FGF23 production and immunoassay measurements. (**a**) After completed transcription and translation, FGF23 can be transferred to two post-translation modification pathways, including O-glycosylation with GALNT3 on Thr178, or phosphorylation by the extracellular serine/threonine protein kinase FAM20C at Ser180. O-glycosylation modification by GALANT3, stabilized form, can prevent intact FGF23 from cleavage. In contrast, phosphorylated FGF23 by FAM20C can be cleaved into N-terminal and C-terminal fragments within the osteocyte/osteoblast. These peptides, including full-length (intact) FGF23, N-terminal fragments, and C-terminal fragments, can be detected in the circulation. (**b**) For C-terminal assays, detecting antibodies bind to C-terminus epitopes to detect both full-length FGF23 and its C-terminal fragments, whereas assays for intact FGF23 use antibodies to detect epitopes surrounding the FGF23 cleavage site for the detection of only full-length FGF23. This figure was generated with publication licensed by BioRender, Toronto, ON, Canada (Agreement number: DV237SONHF, 19 November 2021). Abbreviations: GALNT3, polypeptide N-acetyl galactosaminyltransferase 3; FAM20C, the extracellular protein kinase FAM20C; Ser, Serine; Thr, Threonine.

Since none of the commercially available FGF23 assays have been validated for clinical use, FGF23 is not presently applicable for routine clinical practices. For the measurement of FGF23, four immunoassays are commercially available: Immutopics (1st and 2nd generation, San Clemente, CA, USA), Kainos (Tokyo, Japan), Millipore (Billerica, MA, USA), and DiaSorin (Saluggia, Italy). The majority of assays detect the intact 251 amino acid protein (iFGF23) by simultaneously recognizing epitopes on the N- and C-terminal domains located near the proteolytic cleavage site. Additionally, Immutopics provides an assay that quantifies both iFGF23 and the C-terminal fragment (cFGF23) using two antibodies against two C-terminal epitopes. iFGF23 is measured in picograms per milliliter (pg./mL), with a normal reference range of 11.7–48.6 pg./mL in a healthy individual, whereas cFGF23 is reported in relative units (RU) per milliliter, with a normal reference range of 21.6–91.0 RU/mL [47]. Due to the possibility that iFGF23 may be degraded by protease enzyme or changed after venipuncture, two iFGF23 stability studies discovered decreasing FGF23 levels following an 8-h delay in centrifugation, but no evidence of deterioration after storing processed samples at −80 °C [48]. Biological variability studies in healthy individuals revealed that iFGF23 levels have a diurnal variation that peaks in the early morning and gradually declines during the day [47]. In comparison, the concentrations of cFGF23 could be slightly increased throughout the day [49] and see no significant change after dietary or phosphate intake [50]. Despite the stability and biological variability advantages of cFGF23, cFGF23 assays may be more applicable than iFGF23 assays, particularly for diagnostic and prognostic studies. In contrast, iFGF23 may outperform in representing the biological effects of FGF23 in etiognostic and therapeutic research because the c-terminal fragments might have counter-regulatory effects on the physiologically active FGF23 [51]. The schematic summary of FGF23 production and its measurement are illustrated in Figure 4.

## 12. Conclusions

A pivotal role of FGF23 was found in local and systemic bone remodelling with supraphysiological levels causing abnormal bone formation, although any direct effect on osteoblasts remains unclear as well as a controversial links between FGF23 with osteoclastogenesis and bone resorption. Current evidence from clinical studies indicates that FGF23 could be a risk factor of bone fragility in CKD-MBD, but not a major contributor to age-related osteoporosis. An increased FGF23 level may represent an abnormal state of bone mineral homeostasis, but is not a direct indicator of decreased BMD. Since clinical studies, both in healthy elderly and in patients with impaired renal function, showed that elevated FGF23 levels were an independent risk factor of fragility fracture, a future predictive model for fragility fracture may incorporate FGF23 as a factor to represent bone mineral homeostasis status. FGF23 is putative factor in the fragility of CKD-MBD, less so in age-related bone loss; future elucidation of pathogenesis requires remodelling biomarkers, while gender differences need elucidation with respect to abnormal bone mineral homeostasis and reduced BMD as part of a future model of fragility risk factors.

## Figures and Tables

**Table 1:** The association between FGF23 and bone mineral density and fragility fracture in elderly.

Study Design			Key Findings					Interpretation	Ref.
Patient Group	Age (Year)	Study Base	Serum FGF23 (pg./mL)	OB Activity/Proliferation	OC Activity/Proliferation	BMD/ Bone Architecture	Fragility Fracture		
Men (*n* = 2782)	75.4 ± 3.1	cross- sectional (Sweden)	49 ± 40.8 ^E^	N/A	N/A	↔ BMD ^a (age, smoking, height, weight)^	N/A	FGF23 weakly associated with BMD in elderly men, but there was not clinical and statistical significance after adjustment with potential confounders.	[2]
						↑ BMD ^a (Pi, Ca, PTH, eGFR, 25(OH)2D3)^			
Men (*n* = 3014)	75.4 ± 3.2	prospective cohort	ref. > 57.4 ^E^ (median)	N/A	N/A	N/A	↑ ^a (BMI, FN BMD, eFGR, Pi, PTH, 25(OH)2D3, other fracture risks)^	High circulating FGF23 was an independent risk factor of overall fragility fracture in elderly men.	[29]
		(Sweden)							
Men (*n* = 1680) eGFR > 60	73.7 ± 5.8	prospective case-cohort (USA)	22.4–111.1 ^I^ (quartile 4)	N/A	N/A	↔ BMD	↔ ^a (FN BMD, Pi, PTH, 25(OH)2D3, other fracture risks)^	There was no significant association between BMD or osteoporosis fracture with FGF23 in elderly men who had eFGR > 60.	[28]
Men (*n* = 997)	75.3 ± 3.2	cross- sectional	42.2 (20.6) ^I^	N/A	N/A	↑ LS BMD ^a (age, BMI, cysC, Pi, PTH, 25(OH)2D3, Apo-B/A1 ratio, Iron study)^	N/A	Increasing serum FGF23 level in elderly men was weakly associated with lumbar BMD.	[30]
		(Sweden)							
pre- menopause (*n* = 60)	43.8 ± 5.3	cross- sectional (China)	44.5 ± 9.2 ^E^	ref.	ref.	ref.	N/A	In early post-menopause, estrogen deprivation caused BMD reduction from excessive bone resorption and decreased bone formation. Increased FGF23 might be a compensational response to this process. In post-menopausal women with low bone mass, increasing FGF23 showed a strong negative correlation with BMD.	[1]
early menopause (*n* = 60)	48.6 ± 4.7		76.7 ± 11.6 ^E^	↓ OC ↑↑P1NP	↑ CTX-1	↓ PF BMD ↓ LS BMD	N/A		
late menopause (*n* = 60)	53.4 ± 3.2		29.2 ± 8.6 ^E^	↓ OC ↔P1NP	↑ CTX-1	↓↓ PF BMD ↓↓ LS BMD	N/A		
post menopause with low bone mass (subgroups)									
PF t-score −1 to −2			73.5 ± 9.6 ^E^	N/A	N/A	↓↓ PF BMD ^b^	N/A		
PF t-score <−2			82.5 ± 8.4 ^E^						
LS t-score −1 to −2			75.5 ± 9.7 ^E^	N/A	N/A	↓↓ LS BMD ^b^	N/A		
LS t-score <−2			82.9 ± 9.1 ^E^						
osteoporosis patient (*n* = 82)	64.0 ± 12.7	cross- sectional (Germany)	98 ± 133 ^C^	↔ BALP	N/A	↓ BV/TV ^a (age, BMI, Pi, PTH, 25(OH)2D3, BAP)^	N/A	High FGF23 was associated with reduced trabecular bone micro-architecture in osteoporosis.	[3]
						↓↓ Tb.N ^a (age, BMI, Pi, PTH, 25(OH)2D3, BAP)^			
						↓ Tb.Th ^a (age, BMI, Pi, PTH, 25(OH)2D3, BAP)^			
All genders (*n* = 73)	76.2 ± 8.0	cross- sectional (Japan)	37 (12.7) ^E^	↓ BALP ^b^	↓ P1NP ^b^	↔BMD ^b^	N/A	FGF23 did not show a clinically significant association with BMD and bone remodeling when adjusted for confounders.	[31]
					↔ TRAP5b ^a (eGFR, 25(OH)2D3)^				
post menopause (*n* = 55)	61 ± 1.1	cross- sectional (Romania)	81.2 ± 3.6 ^C^	N/A	↔ CTX-1 ^b^	↓ FN BMD ^a (PTH, 25(OH)2D3, Leptin)^	N/A	Serum FGF23 level was independently associated with decreasing BMD in the femoral neck in post- menopausal women.	[4]

^a^ adjusted by multivariate analysis, ^b^ univariate correlation, ^C^ C-terminal fragment FGF23 (kRU/L) ELISA, ^E^ intact FGF23 two site monoclonal ELISA, ^I^ intact FGF23 polyclonal ELISA; Values are expressed as mean ± SD and median (IQR), ↑↑: very significant increased, ↑: significant increased, ↔: no significant difference, ↓: significant decrease, ↓↓: very significant decrease (within study comparison); Abbreviations: BALP, bone alkaline phosphatase; BMD, bone mineral density; BV/TV, bone volume/trabecular volume; CTX-1, serum c-telopeptide of type 1 collagen; FN, femoral neck, FT, Femoral trochanter; N/A, data not available; OC, serum osteocalcin; P1NP, serum propeptide of type 1 procollagen; PF, proximal femur, LS: lumbar spine; ref., reference (comparison group by univariate analysis), TH, total hip; Tb.N, trabecular number; Tb.Th, trabecular thickness; TRAP5b, serum tartrate-resistant acid phosphatase 5b.

**Table 2:** The association between FGF23 and bone mineral density and fragility fracture in CKD and ESRD patients.

Study Design			Key Findings					Interpretation	Ref.
Patient Group	Age (Year)	Study-Based	Serum FGF23 (pg./mL)	OB Activity/Proliferation	OC Activity/Proliferation	BMD	Fragility Fracture		
post menopause eGFR 45.7 ± 24.1 (*n* = 105)	73.2 (7.7)	cross-sectional (Japan)	49 (37) ^E^ ref. > 56.8 ^E^	N/A	↔NTX ^a (age, BMI, eFGR, Ca, Pi, PTH, 1,25(OH)2D)^	N/A	↑ vertebral ^a (age, eGFR)^	The higher level of FGF23 was associated with vertebral fracture in the elderly with CKD. There was no significant difference between FGF23 and osteogenic biomarkers.	[34]
men	73.7	prospective	22.4–111.1 ^I^	N/A	N/A	N/A	↑↑ non-vertebral ^a (FN BMD, Pi, PTH, 25(OH)2D3, other fracture risks)^	FGF23 elevation had increased the risk of non- vertebral fractures in the subgroup of elderly men with CKD (eFGR < 60).	[28]
eGFR < 60	± 5.8	case-cohort	(quartile 4)						
subgroup, (*n* = 313)		(USA)							
CKD 2–5 (*n* = 142)	67 ± 12	prospective cohort	52.55 ± 55.19 ^E^	N/A	N/A	↔ ^b^	N/A	Serum FGF23 level was not associated with BMD in CKD patients.	[46]
		(France)							
ESRD with MHD	60.6 ± 11.3	cross- sectional	N/A	N/A	N/A	↔ ^b^	N/A	No association between FGF23 and BMD in ESRD patients was found. FGF23 in ESRD patients with osteoporosis was significantly higher than patients without osteoporosis or osteopenia.	[44]
(*n* = 64)		(China)							
Subgroup									
normal (*n* = 10)	55.4 ± 5.0		218.7 ± 28.6 ^E^	ref.	N/A	t-score > −1	N/A		
osteopenia (*n* = 24)	64.4 ± 3.9		235.6 ± 54.4 ^E^	↑BALP	N/A	t-score −1 to −2.5	N/A		
osteoporosis (*n* = 30)	67.4 ± 3.8		296.2 ± 48.6 ^E^	↑↑BALP	N/A	t-score < −2.5	N/A		
KT (*n* = 106) eFGR	40.1 ± 11.1	cross- sectional	25.29 ± 30.81 ^E^	N/A	N/A	↔ ^b^	N/A	No relationship was found between FGF23 and BMD in KT and CKD patients.	[42]
72.6 ± 27.1		(Turkey)							
CKD (*n* = 30) eGFR 65.2 ± 54.6	39.2 ± 11.3		28.86 ± 26.5 ^E^	N/A	N/A	↔ ^b^	N/A		
post menopause (*n* = 102)		prospective case-control (Greece)	N/A	N/A	N/A	↔ ^b^	N/A	Elevated FGF23 was not associated with BMD in ESRD with HD. However, BMD was significantly decreased when compared with health matched controls.	[43]
Subgroups									
ESRD with MHD (*n* = 50)	62 ± 9.6		1027.8 ± 556.7 ^C^	N/A	N/A	↓	N/A		
healthy control (*n* = 52)	59 ± 9.5		100.3 ± 54.7 ^C^	N/A	N/A	ref.	N/A		
ESRD with MHD (*n* = 130)	72 (14)	Prospective nested case-control (Canada)	787 (1430) ^C^	↔ OC ^b^ ↔P1NP ^b^	↔ TRAP5b ^b^ ↔ Sclerostin ^b^	N/A	↑ all fracture ^a (PTH, ferritin, smoking, P1NP)^	C-terminal FGF23 was associated with fracture incidence in ESRD with regular HD patients. There was no significant correlation between FGF23 and bone cell biomarkers.	[35]
ESRD with MHD	53 ± 14.6	cross- sectional	221.9 ± 248.9 ^E^	↔BALP ^b^	↔CTX-1 ^b^	↔ ^b^	↔	FGF23 was significantly increased in ESRD patients with lumbar spine osteo- porosis, but no correlation between BMD and FGF23 was observed. The biomarkers related to bone formation and resorption did not show any difference between normal bone, osteopenia and osteoporosis.	[45]
(*n* = 90)		(Tunisia)							
Subgroups									
LS osteoporosis (*n* = 8)	65.8 ± 10.1		↑ 428.1 ± 275.6 ^E^	↔ BALP	↔ CTX-1	↔ ^b^	N/A		
vs. normal/ osteopenia (ref.)									
TH osteoporosis (*n* = 18)	63.9 ± 11.4		↔ 250.3 ± 250.3 ^E^	↔ BALP	↔ CTX-1	↔ ^b^	N/A		
vs. normal/ osteopenia (ref.)									

^a^ adjusted by multivariate analysis, ^b^ univariate correlation, ^C^ C-terminal fragment FGF23 (kRU/L) ELISA, ^E^ intact FGF23 two site monoclonal ELISA, ^I^ intact FGF23 polyclonal ELISA; Values are expressed as mean ± SD and median (IQR), ↑↑: very significantly increased, ↑: significantly increased, no significant difference, ↓: significantly decreased, ↓↓: very significantly decreased (within study comparison); Abbreviations: all fractures, (hip fractures, other fractures, and vertebral fractures); BMD, bone mineral density; BALP, bone alkaline phosphatase; CKD, chronic kidney disease; CTX-1, serum c-telopeptide of type 1 collagen; ESRD, end-stage renal disease; KT, kidney transplant; TH, total hip; LS, lumbar spine; MHD, maintenance hemodialysis; non-vertebral, hip fracture and other fractures; NTX, urine N-terminal telopeptide; N/A, data not available; OC, serum osteocalcin; P1NP, serum propeptide of type 1 procollagen; ref., reference (comparison group by univariate analysis); TRAP5b, serum tartrate-resistant acid phosphatase 5b.

## Data Availability

Not applicable.

## Supplementary Materials

The following supporting information can be downloaded at: https://www.mdpi.com/article/10.3390/ijms23052500/s1.
