# Supplementary material for: Fibroblast Growth Factor 23 and Osteoporosis: Evidence from Bench to Bedside
Source: Int J Mol Sci. 2022 Feb 24;23(5):2500. doi: 10.3390/ijms23052500 (PMC8909928; doi:10.3390/ijms23052500)
Supplement: Supplementary file 1 [file ijms-23-02500-s001.zip › ijms-1597613-supplementary.pdf]

Table S1. Effects of FGF23 on systemic mineral homeostasis

Model	Experiments/ interventions	Key findings				Interpretation	Ref
		PTH	Vitamin D	Phosphate	Calcium		
Increased FGF23 effects							
Cattle parathyroid cells	cell cultured in FGF23 (R176Q)	↓ mRNA (dose-dependent)	↑ 1α-OHase mRNA (dose-dependent)	N/A	N/A	PTH production had a negative correlation with FGF23, while positively correlating to vitamin D activation in bovine parathyroid cells.	(Krajisnik et al., 2007)
Sabra rat	FGF23 (R176Q) IV	↓ mRNA	↓↓ 1,25$(OH)_2$D	↓	N/A	• FGF23 inhibited PTH production via MAPK pathway.<br>• Excessive FGF23 induced hypophosphatemia and decreased active vitamin D.	(Ben-Dov et al., 2007)
Sabra rat parathyroid cells	FGF23 (R176Q) IV + cell cultured in ERK1/2 inhibitor media	↔ mRNA	N/A	N/A	N/A		
Decreased FGF23 expression							
ICR mice kidney	*Fgf23*$^{-/-}$	↓↓	↑ 1α-OHase mRNA<br>↑ 1,25$(OH)_2$D	↑<br>(+) NaPi-2a IHC proximal tubule<br>↑ NaPi-2a uptake activity of BBMV<br>↑ TmP/GFR	↑	Lack of FGF23 affected PTH secretion, vitamin D activation, and phosphate reabsorption via NaPi-2a.	(Shimada et al., 2004)
	Fgf23$^{-/-}$ + recombinant FGF23 IV	N/A	↓ 1,25$(OH)_2$D	N/A	N/A		
Mice	*Cre*$^{+}$ *VDR*$^{fl/fl}$	↔	↑ 1,25$(OH)_2$D	↑	↔	FGF23 reduction positively correlated with active vitamin D level and phosphate reabsorption, but there was no significant effect on serum PTH and calcium.	(Masuyama et al., 2006)
Mice kidneys			↑ CYP27B1<br>↔ CYP24	↑ NaPi-2a uptake activity of BBMV			
Decreased FGF23 effects							
Mice	Klotho KO (systemic)	↔ urine cAMP/Cr. (PTH activity)	↔ 1,25$(OH)_2D_3$	↑	↑	Lack of Klotho exhibited effects on phosphate and calcium similar to FGF23 absence but did not affect active vitamin D level and PTH activity.	(Kawaguchi et al., 1999)
Mice kidneys	*αKlotho* KO (proximal tubular cells vs systemic)	↓↓	↑↑ 1,25$(OH)_2$D<br>↑↑ CYP27B1 mRNA<br>↓ CYP24A1 mRNA	↑↑<br>↑↑ NaPi-2a mRNA	↑	Klotho and FGFR1-4 in proximal tubules mainly involved FGF23 actions on phosphate reabsorption via NaPi-2a and vitamin D regulation in mice kidney.	(Takeshita et al., 2018)
	Fgfr1-4 KO	↓↓	↑↑ 1,25$(OH)_2$D	↑↑	↑		

Model	Experiments/ interventions	Key findings				Interpretation	Ref
		PTH	Vitamin D	Phosphate	Calcium		
	(proximal tubular cells vs systemic)		↑↑ CYP27B1 mRNA<br>↓ CYP24A1 mRNA	↑↑ NaPi-2a mRNA			

Abbreviations: BBMV: Bush-boarder membrane vesicles; CYP27B1: CYP27B1 expression (regulated hydroxylation of 25(OH)D to its active form); CYP24A1: CYP24A1 expression (regulated vitamin D inactivation); FGF23: Fibroblast growth factor 23; FGF23 (R176Q): Aberrated proteolytic cleavage site FGF23 (excessive activity); *Fgf23*$^{-/-}$: Homogenous FGF23 gene knock-out; *Fgfr* 1-4: Fibroblast growth factor receptor 1-4, IHC: Immunohistochemistry; IV: Intravenous injection, KO: Knock-out; NaPi-2a: Sodium-phosphate co-transporter 2a; MAPK: Mitogen-activated protein kinase; N/A: data not available; TmP: Tubular maximum transport of phosphate; VDR: Vitamin D receptor; 1α-OHase: 1α-hydroxylase; $1,25(OH)_2D$: serum 1,25-dihydroxyvitamin D
↑↑: very significant increased expression, ↑: significant increased expression, ↔: no significant difference, ↓: significant decreased expression, ↓↓: very significant decreased expression, + significant expression (within study comparison)

References

Ben-Dov, I. Z., Galitzer, H., Lavi-Moshayoff, V., Goetz, R., Kuro-O, M., Mohammadi, M., Sirkis, R., Naveh-Many, T. & Silver, J. 2007. The parathyroid is a target organ for FGF23 in rats. *The Journal of clinical investigation,* 117**,** 4003-4008. ((https://doi.org/10.1172/JCI32409)

Kawaguchi, H., Manabe, N., Miyaura, C., Chikuda, H., Nakamura, K. & Kuro-O, M. 1999. Independent impairment of osteoblast and osteoclast differentiation in klotho mouse exhibiting low-turnover osteopenia. *The Journal of clinical investigation,* 104**,** 229-237. ((https://doi.org/10.1172/jci5705)

Krajisnik, T., Björklund, P., Marsell, R., Ljunggren, O., Akerström, G., Jonsson, K. B., Westin, G. & Larsson, T. E. 2007. Fibroblast growth factor-23 regulates parathyroid hormone and 1alpha-hydroxylase expression in cultured bovine parathyroid cells. *Journal of Endocrinology,* 195**,** 125-31. ((https://doi.org/10.1677/joe-07-0267)

Masuyama, R., Stockmans, I., Torrekens, S., Van Looveren, R., Maes, C., Carmeliet, P., Bouillon, R. & Carmeliet, G. 2006. Vitamin D receptor in chondrocytes promotes osteoclastogenesis and regulates FGF23 production in osteoblasts. *The Journal of clinical investigation,* 116**,** 3150-3159. ((https://doi.org/10.1172/JCI29463)

Shimada, T., Kakitani, M., Yamazaki, Y., Hasegawa, H., Takeuchi, Y., Fujita, T., Fukumoto, S., Tomizuka, K. & Yamashita, T. 2004. Targeted ablation of Fgf23 demonstrates an essential physiological role of FGF23 in phosphate and vitamin D metabolism. *The Journal of clinical investigation,* 113**,** 561-568. ((https://doi.org/10.1172/JCI19081)

Takeshita, A., Kawakami, K., Furushima, K., Miyajima, M. & Sakaguchi, K. 2018. Central role of the proximal tubular αKlotho/FGF receptor complex in FGF23-regulated phosphate and vitamin D metabolism. *Scientific Reports,* 8**,** 6917. ((https://doi.org/10.1038/s41598-018-25087-3)

Table S2. Regulation of FGF23 and its effects on osteoblasts, osteoclasts, and bone formation

Models	Experiments/ intervention	Key findings				Interpretation	Ref
		FGF23 mRNA	OB activity/ proliferation	OC activity/ proliferation	Bone formation		
Wistar rat calvaria cells (osteoblast)	none	↓<br>↑cell protein	↑ BSP<br>↑ OC<br>↑ ALP	N/A	↑ ALP positive bone nodule<br>↑ OPN	• FGF23 was increased during peaked osteoblast development and bone nodule formation but might suppressed matrix mineralization caused by elevated OPN.<br>• Increasing FGF23 was regulated by$1,25(OH)_2D_3$ in deterministic manner and might inhibited matrix mineralization.	(Yoshiko et al., 2007)
	β-GP	↑ 48 hr.<br>↓ 96 hr.	N/A	N/A	↑ matrix mineralization at 48 hr.		
	β-GP + $1,25(OH)_2D_3$ (0.1-10 nM)	↑<br>(dose-dependent)	N/A	N/A	↓ matrix mineralization		
Mouse chondrocyte /osteoblasts (co-cultured)	*Cre*$^{+}$ *VDR*$^{fl/fl}$ +$1,25(OH)_2D_3$ (0.1-10 nM)	↓↓	↔ OPG<br>↔ OC<br>↔ Runx2	Absent RANKL mRNA	N/A	• Chondrocyte VDR and $1,25(OH)_2D_3$ action regulated FGF23 secretion via osteoblasts.<br>• $1,25(OH)_2D_3$ action via chondrocyte VDR strongly induced osteoclastogenesis.<br>• Increased bone volume and trabecular bone ratio might be caused by decreased osteoclastogenesis from lack of VDR signaling.	(Masuyama et al., 2006)
	Cre^{-} *VDR*$^{fl/fl}$ +$1,25(OH)_2D_3$ (0.1-10 nM)	↔	↔ OPG<br>↔ OC<br>↔ Runx2	↑ RANKL mRNA	N/A		
Mouse osteoblasts	*Cre*$^{+}$ *VDR*$^{fl/fl}$ +$1,25(OH)_2D_3$ (0.1-10 nM)	↔	N/A	N/A	N/A		
	Cre^{-} *VDR*$^{fl/fl}$ +$1,25(OH)_2D_3$ (0.1-10 nM)	↔	N/A	N/A	N/A		
Mouse embryogenic chondrocytes/ osteoblasts (co-cultured)	*Cre*$^{+}$ *VDR*$^{fl/fl}$ + $1,25(OH)_2D_3$ (0.1-10 nM)	↓↓	N/A	N/A	N/A		
	Cre^{-} *VDR*$^{fl/fl}$ + $1,25(OH)_2D_3$ (0.1-10 nM)	↔	N/A	N/A	N/A		
Mouse	*Cre*$^{+}$ *VDR*$^{fl/fl}$	N/A	N/A	N/A	↑ BV/TV<br>↔ BMD		
	Cre^{-} *VDR*$^{fl/fl}$	N/A	N/A	N/A	Ref.		
Femurs from ICR mice	*Fgf23*$^{-/-}$	Absent	↑↑ ALP mRNA	N/A	Absent	FGF23 was a crucial factor for bone matrix mineralization.	(Shimada et al., 2004)
	Fgf23$^{+/-}$	↔	↔ ALP mRNA	N/A	↔ MAR		
Femurs from mice	*Phex*$^{-/-}$	↑	N/A	N/A	↓ BMD	• Lack of DMP1 and PHEX caused excess FGF23 via FGFR1 signaling without the synergistic effect and bone mineralization defect.<br>• PHEX and DMP1 had a direct effect on bone mineralization.	(Martin et al., 2011)
	Dmp$^{-/-}$	↑↑	N/A	N/A	↓ BMD		
	Phex$^{-/-}$ /*Dmp*$^{-/-}$	↑↑	N/A	N/A	↓ BMD		
Mice MSC	*Wild type* + SU5402	↓	↔ Runx2	N/A	↓ Matrix mineralization		
	Phex$^{-/-}$ + SU5402	↔	↔ Runx2	N/A	↓ Matrix mineralization		
	Dmp$^{-/-}$ + SU5402	↔	↔ Runx2	N/A	↓ Matrix mineralization		

Models	Experiments/ intervention	Key findings				Interpretation	Ref
		FGF23 mRNA	OB activity/ proliferation	OC activity/ proliferation	Bone formation		
Human MSC-derived osteoclast	FGF23 (1-10 ng/ml)	N/A	N/A	↓ TRAP+MNCs (dose-dependent) (day 0)<br>↔ TRAP+MNCs (day 3, day 6)	↑ resorbed area per well/ per osteoclasts	FGF23 has biphasic effects on osteoclast physiology, inhibiting osteoclastogenesis while marginally increased osteoclast activity.	(Allard et al., 2015)
	FGF23 (1-10 ng/ml) + FGFR inhibitor (250 nM)	N/A	N/A	↔ TRAP+MNCs (day 0)	N/A		
Mice	*αKlotho* KO (systemic)	↑↑	N/A	N/A	↓ BMD	The effect of FGF23 mainly contributed to the action via αKlotho/FGFR1-4 complex expressed in proximal tubules.	(Takeshita et al., 2018)
	αKlotho KO (proximal tubular cells)	↑↑	N/A	N/A	↓ BMD		
	Fgfr1-4 KO (proximal tubular cells)	↑↑	N/A	N/A	↓ BMD		
Mouse MSC	*αKlotho KO* + FGF23 (500 - 2,000 pg/ml)	N/A	↔	N/A	↓ osteoid-nodule formation	Physiologic level of FGF23 induced osteoblastic activity and differentiation of mouse bone marrow MSC and matrix mineralization inhibition by OPN upregulation via FGF23-Klotho-FGFRs complex.	(Li et al., 2013)
	Wild type + FGF23 (500 - 2,000 pg/ml)	N/A	↑ OC (dose-dependent)<br>↑ALP (dose-dependent)	N/A	↑ OPN (dose-dependent)		
	Wild type + FGF23 (500 - 2,000 pg/ml) + SU 5402	N/A	↓ Runx2<br>↓ OC<br>↓ ALP	N/A	↓ OPN		
Mice	*VDR*$^{\Delta/\Delta}$	↔ serum	N/A	N/A	↔ C.BMD<br>↔ T.BMD<br>↔ PPi /↔ TNAP<br>↓ OPN	• FGF23 controlled bone mineralization by indirectly decrease OPN via suppression of TNAP in a 1,25(OH)2D3 and Klotho-independent manner.<br>• OPN upregulation in osteoblast was directly controlled via VDR, whereas TNAP was directly suppressed by FGF23-FGFR3 action.<br>• FGF23 suppressed OPN secretion indirectly through changes in the extracellular<br>• inorganic phosphate concentration caused by inhibiting TNAP enzyme activity.	(Murali et al., 2016)
	Klotho$^{-/-}$	↑↑ serum	↔ ALP	N/A	↓ C.BMD<br>↓ T.BMD<br>↑ PPi /↓ TNAP<br>↑↑ OPN		
	Klotho$^{-/-}$ */VDR*$^{\Delta/\Delta}$	↔ serum	N/A	N/A	↔ C.BMD<br>↔ T.BMD<br>↓ PPi /↑ TNAP<br>↓ OPN		
	Fgf23$^{-/-}$	N/A	↔ ALP	N/A	↓ C.BMD<br>↓ T.BMD<br>↑ PPi /↑ TNAP<br>↑ OPN		
	Fgf23$^{-/-}$ */VDR*$^{\Delta/\Delta}$	N/A	N/A	N/A	↓ C.BMD<br>↓ T.BMD<br>↓ PPi /↑↑ TNAP<br>↑ OPN		

Models	Experiments/ intervention	Key findings				Interpretation	Ref
		FGF23 mRNA	OB activity/ proliferation	OC activity/ proliferation	Bone formation		
Mice osteoblast	*Wild type* + 1,25(OH)$_2$D$_3$ (10-100 nM)	N/A	N/A	N/A	↑ OPN (dose-dependent) ↔ TNAP	• Supraphysiologic level of FGF23 suppressed osteoblastic activity in dose-dependent manner and Klotho-independent.	
Mice osteoblast	*VDR*$^{\Delta/\Delta}$ + 1,25(OH)$_2$D$_3$ (10-100 nM)	N/A	N/A	N/A	↔ OPN ↔ TNAP		
Mice osteoblast	*Wild type* + rFGF23 (10 - 100ng)	N/A	↓ ALP	N/A	↔ OPN		
	Klotho$^{-/-}$ + rFGF23 (10 - 100ng)	N/A	↓ ALP (dose-dependent)	N/A	↓ TNAP (dose-dependent)		
	Wild type + anti-TNAP	N/A	N/A	N/A	↑ PPi ↑ OPN		
	Wild type + anti-FGF23	N/A	N/A	N/A	↓ PPi ↑↑ OPN		
	Wild type + anti-FGF23 + anti-TNAP	N/A	N/A	N/A	↑ PPi ↑ OPN		

Abbreviations: ALP: Alkaline phosphatase; β-GP: β-glycerophosphate; BMD: Bone mineral density; BSP: Bone sialoprotein mRNA; C.BMD: Cortical bone mineral density; Dmp–/–: Dentin Matrix Acidic Phosphoprotein 1 gene knock-out; Fgfr 1-4: Fibroblast growth factor receptor 1-4; FGF23: Fibroblast growth factor 23; Fgf23–/–, Homogenous FGF23 gene knock-out; Fgf23+/–: Homogenous FGF23 gene knock-out; KO, Knock-out; MAR, Mineral apposition rate; MSC, Mesenchymal stem cell; MNCs: Multinucleated osteoclasts; N/A: data not available; OPG: Osteoprotegerin mRNA; OPN: Osteopontin mRNA; OC: Osteocalcin mRNA; Phex–/– : Phosphate Regulating Endopeptidase Homolog X-Linked gene knock-out; PPi: Pyrophosphate; RANKL: Receptor activator of nuclear factor kappa-B ligand; Runx2: Runt-related transcription factor 2; SU 5402, specific tyrosine kinase inhibitor for FGFRs; T.BMD, Trabecular bone mineral density; TNAP: Tissue nonspecific alkaline phosphatase; 1,25(OH)2D3: 1,25-dihydroxyvitamin D

↑↑: very significant increased expression, ↑: significant increased expression, ↔: no significant difference, ↓: significant decreased expression, ↓↓: very significant decreased expression (within study comparison)

References

Allard, L., Demoncheaux, N., Machuca-Gayet, I., Georgess, D., Coury-Lucas, F., Jurdic, P. & Bacchetta, J. 2015. Biphasic Effects of Vitamin D and FGF23 on Human Osteoclast Biology. *Calcif Tissue Int,* 97**,** 69-79. ((https://doi.org/10.1007/s00223-015-0013-6)

Li, Y., He, X., Olauson, H., Larsson, T. E. & Lindgren, U. 2013. FGF23 affects the lineage fate determination of mesenchymal stem cells. *Calcified Tissue International,* 93**,** 556-64. ((https://doi.org/10.1007/s00223-013-9795-6)

Martin, A., Liu, S., David, V., Li, H., Karydis, A., Feng, J. Q. & Quarles, L. D. 2011. Bone proteins PHEX and DMP1 regulate fibroblastic growth factor Fgf23 expression in osteocytes through a common pathway involving FGF receptor (FGFR) signaling. *The FASEB Journal,* 25**,** 2551-62. ((https://doi.org/10.1096/fj.10-177816)

Masuyama, R., Stockmans, I., Torrekens, S., Van Looveren, R., Maes, C., Carmeliet, P., Bouillon, R. & Carmeliet, G. 2006. Vitamin D receptor in chondrocytes promotes osteoclastogenesis and regulates FGF23 production in osteoblasts. *The Journal of clinical investigation,* 116**,** 3150-3159. ((https://doi.org/10.1172/JCI29463)

Murali, S. K., Roschger, P., Zeitz, U., Klaushofer, K., Andrukhova, O. & Erben, R. G. 2016. FGF23 Regulates Bone Mineralization in a 1,25(OH)2D3 and Klotho-Independent Manner. *Journal of Bone and Mineral Research,* 31**,** 129-142. ((https://doi.org/10.1002/jbmr.2606)

Shimada, T., Kakitani, M., Yamazaki, Y., Hasegawa, H., Takeuchi, Y., Fujita, T., Fukumoto, S., Tomizuka, K. & Yamashita, T. 2004. Targeted ablation of Fgf23 demonstrates an essential physiological role of FGF23 in phosphate and vitamin D metabolism. *The Journal of clinical investigation,* 113**,** 561-568. ((https://doi.org/10.1172/JCI19081)

Takeshita, A., Kawakami, K., Furushima, K., Miyajima, M. & Sakaguchi, K. 2018. Central role of the proximal tubular αKlotho/FGF receptor complex in FGF23-regulated phosphate and vitamin D metabolism. *Scientific Reports,* 8**,** 6917. ((https://doi.org/10.1038/s41598-018-25087-3)

Yoshiko, Y., Wang, H., Minamizaki, T., Ijuin, C., Yamamoto, R., Suemune, S., Kozai, K., Tanne, K., Aubin, J. E. & Maeda, N. 2007. Mineralized tissue cells are a principal source of FGF23. *Bone,* 40**,** 1565-1573. ((https://doi.org/10.1016/j.bone.2007.01.017)
